# Structural snapshots of the glucose-6-phosphate/phosphate exchange cycle

**DOI:** 10.1371/journal.pbio.3003913

**Published:** 2026-07-29

**Authors:** Lin Kevin Qi, Cheng Shen, Motoyuki Hattori

**Affiliations:** State Key Laboratory of Genetics and Development of Complex Phenotypes, Collaborative Innovation Center of Genetics and Development, Department of Physiology and Neurobiology, School of Life Sciences, Fudan University, Shanghai, China

## Abstract

The human glucose-6-phosphate transporter (SLC37A4) mediates the translocation of G6P into the ER. Two recent studies in PLOS Biology report its structures and, together with other reports, illuminate the glucose-6-phosphate/phosphate exchange cycle and its inhibition mechanism.

## Teaser

SLC37A4 mediates glucose-6-phosphate transport for glucose homeostasis. Two recent studies in PLOS Biology report its structures and, together with other reports, illuminate the glucose-6-phosphate/phosphate exchange cycle and its inhibition mechanism.

Between meals, the liver must release glucose produced inside the endoplasmic reticulum (ER) to maintain blood glucose levels. Yet its immediate precursor, cytoplasmic glucose-6-phosphate (G6P), is charged and cannot simply diffuse across the ER membrane. SLC37A4, also known as the glucose-6-phosphate transporter (G6PT), solves this problem by moving G6P into the ER, where the enzyme glucose-6-phosphatase removes its phosphate to produce free glucose. A key mechanistic question is how this transporter recognizes a charged sugar phosphate without becoming a nonspecific anion pathway. Two recent studies in PLOS Biology by Zhang and colleagues and Li and colleagues report structures of human SLC37A4 [1,2]. Four related structural studies were also published in other journals in the order of Xia and colleagues [3], Zhou and colleagues [4], Wang and colleagues [5], and Chen et al. [6]. Together, these six reports provide a structural basis for the transport cycle and inhibition mechanism of SLC37A4 [1–6].

In 1997, mutations found in patients with glycogen storage disease type Ib (GSD-Ib) linked a newly identified ER membrane protein, later known as SLC37A4, to the putative glucose-6-phosphate translocase [7]. This rare inherited disorder causes life-threatening disturbances in glucose homeostasis and immune function, making SLC37A4 clinically important. Functional studies had confirmed that SLC37A4 carries G6P across the ER membrane, and showed that the disease-associated R28H variant severely disrupts this transport [8]. Reconstitution experiments later demonstrated that SLC37A4 exchanges cytoplasmic G6P entering the ER lumen for inorganic phosphate (Pi), moving back to the cytoplasm [9]. This exchange model established SLC37A4 as a G6P/Pi exchanger [10]. However, a detailed understanding of the transport cycle required structural information on where ligands bind and how conformational changes gate access across the ER membrane.

The six recent structural studies have reported apo, G6P- or G6P-analogue-bound, Pi-bound, and inhibitor-bound structures of human SLC37A4 in cytoplasm-facing and ER lumen-facing conformations [1–6]. SLC37A4 follows an alternating-access mechanism common to major facilitator superfamily (MFS) transporters [11]. A central transmembrane cavity is alternately exposed to the cytoplasmic side and the ER lumen side, allowing substrate movement without opening a continuous pore across the membrane [1–6]. Recent G6P- or G6P-analogue-bound structures and AlphaFold3 substrate-binding models place the substrate in a central electropositive cavity, supporting a conserved positively charged pocket for phosphorylated sugars [1–6]. The phosphate group sits in a positively charged pocket shaped by Arg28, Lys29, Lys64, and Lys240, while the sugar ring is stabilized by polar and aromatic amino acid residues [1–6]. This arrangement helps explain how SLC37A4 recognizes a sugar phosphate rather than binding anions nonspecifically. Interestingly, Xia et al.’s earlier G6P-bound structure places the glucose ring closer to the sugar-pocket contacts, whereas Li et al.’s substrate appears in a more loosely engaged, possibly release-like orientation, with the phosphate group still held near the basic pocket [2,3]. The recent structures, therefore, agree on the central cavity but differ in the modeled local ligand placement. This possible intermediate may help provide a more detailed understanding of the transport cycle.

**Fig 1:**
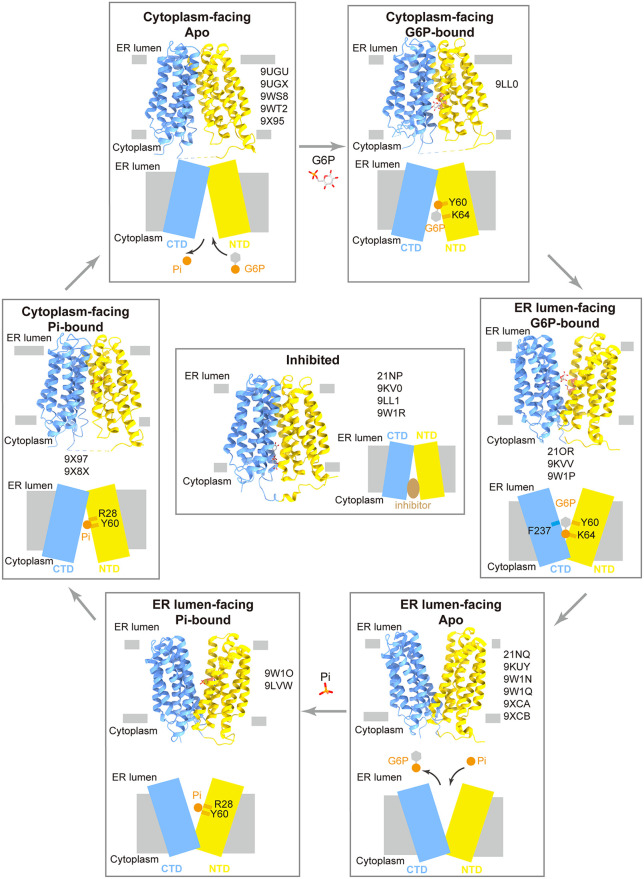
Transport cycle and inhibition model of SLC37A4/G6PT. Available structures from several independent studies suggest that SLC37A4/G6PT achieves alternating-access to the cytoplasm and the endoplasmic reticulum (ER) lumen through rigid-body rotation between the N-domain (yellow) and C-domain (blue). The transport cycle begins with a cytoplasm-facing open conformation, in which the transporter exposes a solvent-accessible cavity with an electropositive surface suitable for attracting negatively charged G6P. G6P binding is proposed to promote a conformational transition from a cytoplasm-facing state toward an ER lumen-facing state, possibly through a putative occluded state. This conformational change lowers G6P affinity, promoting the release of G6P into the ER lumen. After inorganic phosphate (Pi) is loaded, the transporter switches back to a cytoplasm-facing conformation, releasing Pi toward the cytoplasm and completing antiport. Inhibitors such as chlorogenic acid (CHA/CGA) and S-4048 block transport by occluding the substrate-binding pocket and locking the transporter in a cytoplasm-facing state. Representative PDB IDs corresponding to each state are indicated.

After G6P reaches the ER lumen, glucose-6-phosphatase hydrolyzes it to glucose and Pi. Pi can then return through SLC37A4 as the counter-substrate, resetting the carrier for another G6P molecule [9]. Recent structures have now trapped Pi in the electropositive carrier core for both lumen-facing and cytoplasm-facing states [4–6]. Together, the ER lumen-facing and cytoplasm-facing structures from the recent studies provide a structural framework for the transport cycle (Fig 1) [1–6]. Notably, although an occluded state is included in the proposed transport cycle, this state has not yet been directly captured structurally.

By mapping disease-associated residues onto the G6P-binding site, the structures provide a framework for interpreting how disease-associated variants affect substrate recognition. In Li’s functional test, G6P stabilized wild-type SLC37A4 in a thermal-shift assay, whereas substitutions in pocket residues, including Arg28 and Lys64, reduced this stabilization and lowered G6P uptake [2]. Because Arg28 lies in the basic phosphate-contact region, these results point to weakened phosphate recognition as one cause of the R28H transport defect [2,8]. More broadly, these structures and related variant maps help generate mechanistic hypotheses for disease-associated mutations, distinguishing variants that may weaken substrate recognition from those that restrict gate motions, destabilize the fold, or act through still-unresolved structural effects [1–6].

Chlorogenic acid (CHA) is useful not simply because it inhibits SLC37A4, but because it traps the transporter at an interpretable point in its cycle. Earlier transport experiments had shown that CHA blocks both G6P [8] and Pi flux, making it a probe of the exchange mechanism [9]. Recent inhibitor-bound structures show how inhibitors, including CHA, bind from the cytoplasmic side, holding SLC37A4 in a cytoplasm-facing state with the ER lumen gate closed [2–4,6]. The inhibitor-bound structures, therefore, give a structural explanation for how these inhibitors can stabilize cytoplasmic access and prevent the conformational changes needed for exchange.

With various functional states now captured across several studies [1–6], recent structures provide a structural framework for the G6P/Pi exchange cycle and its inhibition mechanism. These structures define targets for future mechanistic studies, including substrate-binding sites, conformationally mobile regions, and inhibitor-stabilized access states. Approaches such as single-molecule analyses could further connect these static snapshots to transport dynamics by revealing state populations, transition kinetics, and transient or off-pathway conformations. These structural insights may also guide rational modulator design by revealing multiple conformationally distinct substrate- and inhibitor-associated pockets that could be used to tune SLC37A4 transport activity. This structural framework represents an important shift from earlier structure-prediction-based approaches, particularly given prior interest in G6PT inhibition as a potential strategy for type 2 diabetes and tumor metastasis [1–6].
